# Multidisciplinary Management of Invasive Basal Cell Carcinoma With Intracranial Invasion: A Rare Case Report

**DOI:** 10.7759/cureus.50938

**Published:** 2023-12-22

**Authors:** Plamen Penchev, Petar-Preslav Petrov, Kiril Ivanov, Simeon Bashev, Bogomil Iliev

**Affiliations:** 1 Department of Medicine, Medical University of Plovdiv, Plovdiv, BGR; 2 Department of Anatomy, Histology and Embryology, Medical University of Plovdiv, Plovdiv, BGR; 3 Department of Medicine, Medical University Sofia, Sofia, BGR; 4 Department of Neurosurgery, Medical University of Varna, Varna, BGR; 5 Clinic of Neurosurgery, University Hospital “Saint Marina”, Varna, BGR

**Keywords:** neuro-oncology, ­skin cancer, plastic surgery, skull reconstruction, ct scan, basal cell carcinoma, case report

## Abstract

The most prevalent kind of skin cancer is basal cell carcinoma (BCC). BCC invasion of the brain occurs quite rarely. Reconstruction approaches along with surgical excision are the gold standard for treating BCC. In this case, we describe a 75-year-old female patient with highly invasive BCC of the head with subdural invasion. The patient underwent surgery in 2022 in another neurosurgery clinic due to BCC of the head, frequent infection of the skin, and involvement of bone structures by the tumor. The patient presented in 2023 to the neurosurgery clinic at Saint Marina University Hospital with cephalgia, right-side hemiparesis, and a 10 x 10 cm skin defect. On a CT scan, we discovered an invasion of the parietal bones of the skull with an extension to the left subdural space. A craniectomy was performed under general anesthesia, along with hard resection to clear the margins of the BCC that had penetrated the cranial bone. Following the resection of the BCC, reconstruction of the skin defect was performed by a plastic surgeon. Consequently, a satisfactory cosmetic outcome was achieved. Postoperative complications were not observed. The patient was followed up for six months.

## Introduction

The non-melanocytic epithelial skin tumor known as basal cell carcinoma (BCC) develops from the basal layer cells of the epidermis [1]. Despite having little evidence of spreading, BCC is a dangerous medical pathology because of its localized destruction and infiltration [2]. The head, face, and scalp are the most often afflicted locations, and exposure to UV radiation is the primary risk factor [2,3]. Recent studies show that the incidence increases quickly, as the lifetime risk reaches 30% [4]. Galea aponeurotica, periosteum, calvaria, epidural and subdural spaces, and the underlying brain parenchyma can all be invaded by the tumor in certain extremely advanced instances; however, intracranial invasion is extremely rare with an estimated incidence of 0.03% [5,6]. This report aims to present a rare case of BCC, highlighting the need to treat it with a multidisciplinary approach.

## Case presentation

**Figure 1:**
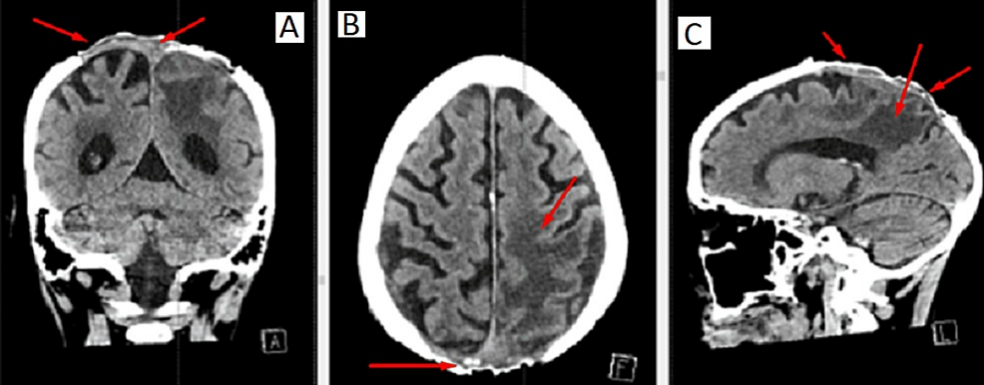
Preoperative CT scan showing evidence of invasion of an extra-axial neoplastic process in both parietal bones and subdural space. Vasogenic edema in the left and right parietal lobes. A: Coronal plane. B: Axial plane. C: Sagittal plane.

We present the clinical case of a 75-year-old female patient who presented to the neurosurgery clinic of Saint Marina University Hospital with cephalgia, right-sided hemiparesis, and a 10 x 10 cm skin defect. A CT scan of the head showed an invasion of the parietal bones of the skull with an extension to the left subdural space (Figure 1). The patient was operated on in another neurosurgical clinic in 2022. As the tumor formation was not resected hard into clear margins, they were not able to perform a skin reconstruction. This resulted in a large defect with a recurrence of the tumor involving the underlying cranial bones and reaching the subdural space.

**Figure 2:**
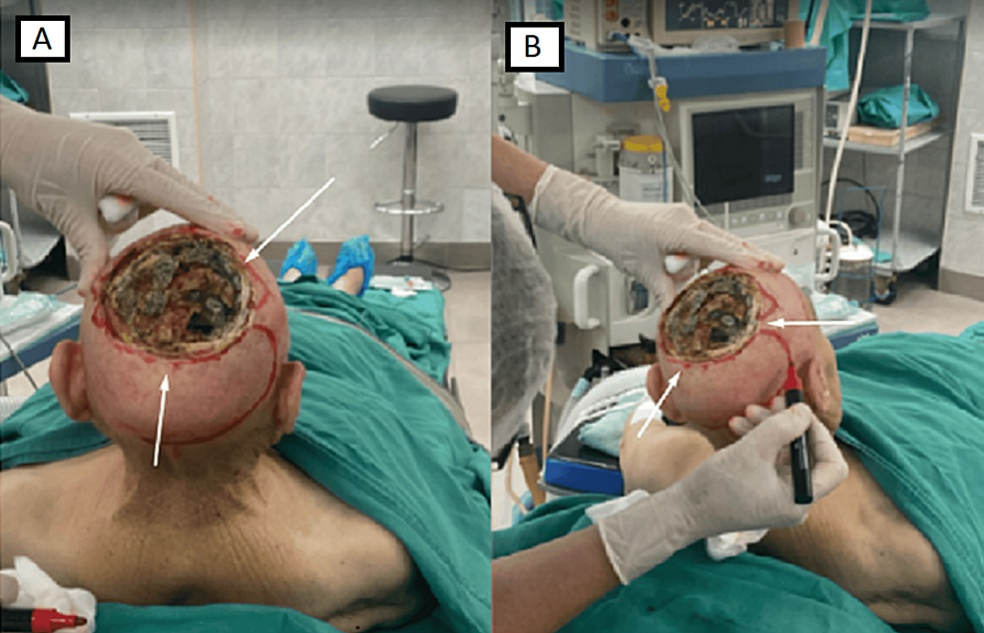
Intraoperative photography. Мarking the skin lamps for skin reconstruction; 10 x 10 cm skin and bone defect in both parietal bones with extension to the subdural space. Invasion and penetration of neoplastic processes to the deep layers of the dura. А: Intraoperative photography, back view. B: Intraoperative photography, lateral view.

Under general inhalation anesthesia, an operative treatment was performed. As it was a recurrent BCC with a highly aggressive nature and extensive intracranial invasion, a 1 cm hard resection was performed, following which the borders were examined pathoanatomically. As a result, we achieved clear surgical margins. Afterward, a revision of the subdural space was performed (Figure 2). Pathology confirmed the diagnosis of sclerosing basal cell carcinoma. Microscopically, it revealed atypical basaloid epithelium set in a densely fibrotic stroma.

**Figure 3:**
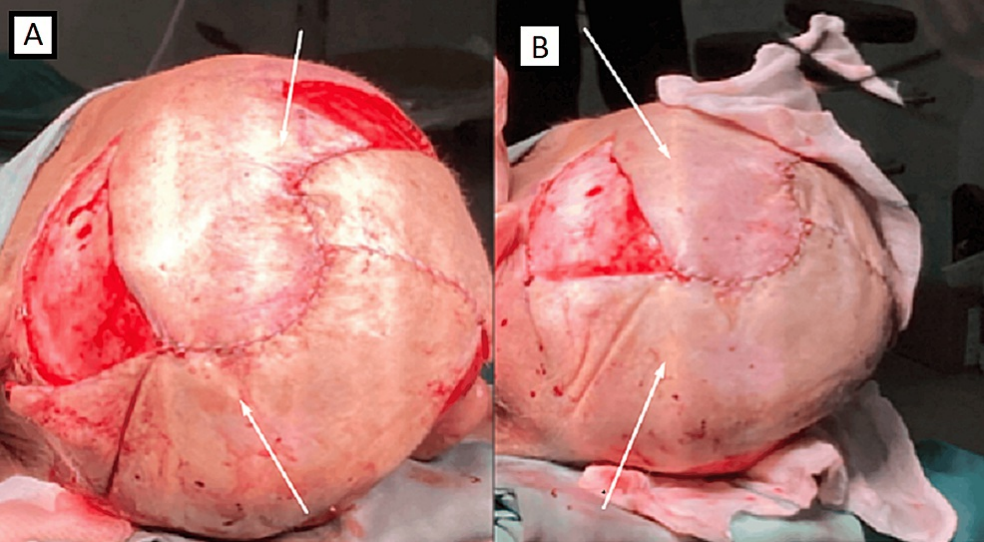
Intraoperative photography. Reconstruction of the skin defect showing good cosmetic effect. А: Intraoperative photography, back view. B: Intraoperative photography, lateral view.

After the excision of the basal cell carcinoma, hermetization and plasty of the dura were performed with a dural patch graft. The reconstruction of the skin defect was performed by a plastic surgeon by rotation of a skin flap and a tissue graft taken from the right thigh. As a result, a good cosmetic result was achieved (Figure 3).

The patient was discharged on postoperative day five in satisfactory condition and was followed up by a plastic surgeon until the plasty was intercepted. In the sixth month, a positron emission tomography-computed tomography scan was performed by an oncologist, with no evidence of metastases or recurrence.

## Discussion

The most prevalent skin malignancy is BCC, which is seen in 93% of cases in sun-exposed regions such as the head and neck. BCC originates from the exterior sheets of hair follicles and/or the basal cells of the epidermis [1,2]. The literature describes various BCC morphologies, including solid, cystic, adenoid, keratotic, infiltrating, pigmented, and sclerosing [6]. In our case, the pathological result confirmed that the diagnosis was sclerosing basal cell carcinoma. Microscopically, it shows strands of atypical basaloid epithelium set in a densely fibrotic stroma. Sclerosing BCC frequently exhibits extensive invasion with high recurrence rates [6].

**Figure 4:**
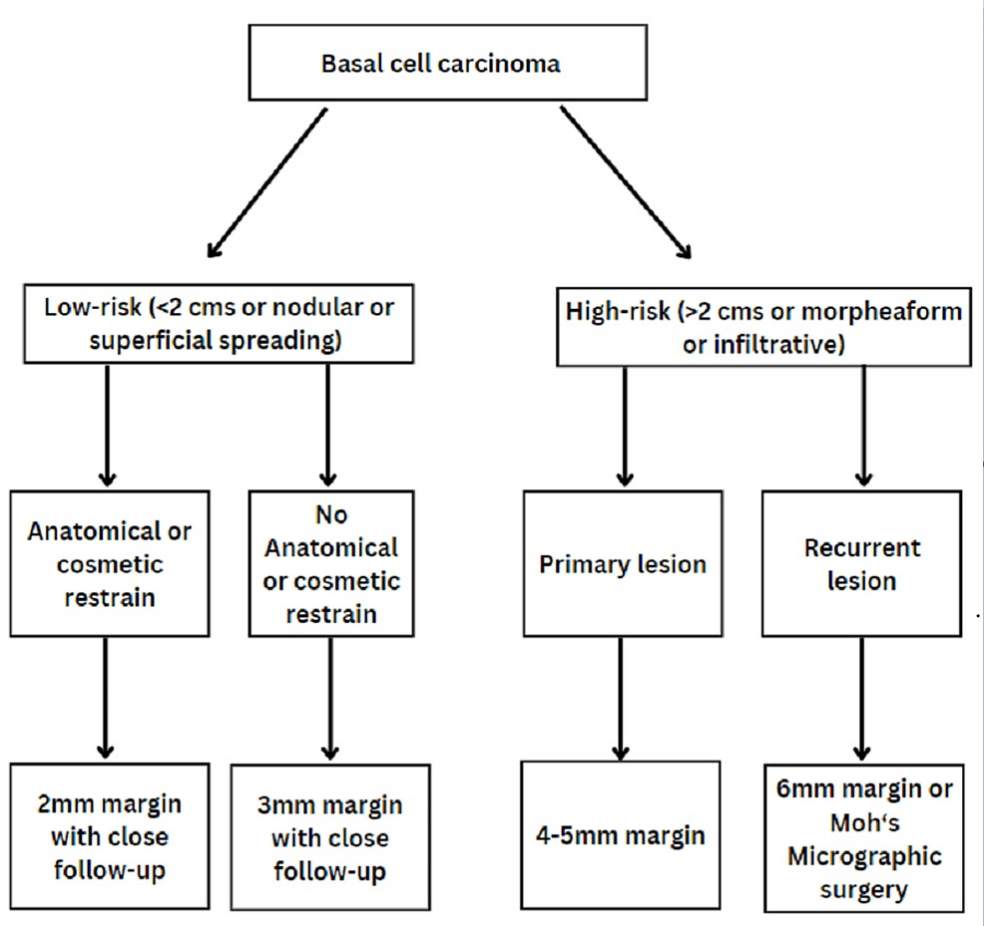
Surgical algorithm for the management of basal cell carcinoma.

The most recent surgical algorithm reported in the literature is by Quazi et al. (Figure 4) [7]. According to their recommendations, for primary high-risk lesions, a 4-5 mm resection margin is required, and for recurrent high-risk lesions, a 6 mm resection margin is required or performing Moh’s micrographic surgery. In our case, as it was a recurrent BCC with a highly aggressive nature and extensive intracranial invasion, a 1 cm hard resection was performed, following which the borders were examined pathoanatomically. As a result, we achieved clear surgical margins. According to Quazi et al., the adequate treatment of invasive BCC is complete resection with clear margins. However, a microscopic invasion of the tumor can occur beyond the clear margins [7].

Hard resection and strict margin control are required during the surgical excision of high-risk BCC [7]. In our case, we performed a hard resection by 1 cm and the pathological borders were examined. As a result, we achieved clear surgical margins. Although BCC in the head region is frequently observed, the risk of involvement of the skull and intracranial structures is very rare, with an estimated frequency of 0.03% [7]. The differential diagnoses include trichoepithelioma, sebaceoma, metastatic process and osteomyelitis, and microcystic adnexal carcinoma [5-7].

A tumor greater than 10 cm is referred to as giant basal cell carcinoma (GBCC), according to recent studies [5,6]. Nonetheless, for tumors larger than 5 cm, some studies refer to them as GBCC [5-7]. We correlate these claims as the tumor bed in our case was 10 x 10 cm. The original tumor size, more than two recurrences despite appropriate therapy, and tumor extension to any extracutaneous tissue are the primary criteria for GBCC [7,8]. According to Vico et al., 1 cm is the threshold at which malignant behavior shifts [9]. Furthermore, a significant and persistent BCC in the head region should raise suspicion for cerebral invasion.

Even in cases when there is no neurological deficit, MRI and CT scans are necessary for a conclusive diagnosis [10-12]. In our case, because of the highly invasive BCC which was suspicious for intracranial invasion, we performed a CT scan which confirmed that the tumor process had extended to the subdural space. According to Kwon et al., the best course of action in these situations is excision of the scalp along with craniectomy and hard resection to clear margins [13].

## Conclusions

For invasive BCC, aggressive tumor excision combined with skin flaps and skin grafting seems to be a practical and effective therapy option. Furthermore, for patients, reconstructive surgery is crucial in terms of appearance to achieve good cosmetic effects. A multidisciplinary approach has a decisive importance in the treatment of this disease.
